# Commentary: Changes in *Bacillus* Spore Small Molecules, rRNA, Germination, and Outgrowth after Extended Sublethal Exposure to Various Temperatures: Evidence that Protein Synthesis Is Not Essential for Spore Germination

**DOI:** 10.3389/fmicb.2016.02043

**Published:** 2016-12-26

**Authors:** Lior Sinai, Sigal Ben-Yehuda

**Affiliations:** Department of Microbiology and Molecular Genetics, Institute for Medical Research Israel-Canada, The Hebrew University-Hadassah Medical School, The Hebrew University of JerusalemJerusalem, Israel

**Keywords:** *Bacillus subtilis*, spores, germination, dormancy, spore revival

Dormant bacterial spores can rapidly revive and resume a vegetative life form once nutrients become available (Stragier and Losick, 1996; Setlow, 2003). The earliest revival event, termed germination, holds the key to understanding this rapid conversion from a dormant into an active cell. During germination, the spore undergoes release of dipicolinic acid (DPA), rehydration, cortex hydrolysis, and coat disassembly. This phase is accompanied by transition from a phase-bright spore to a phase-dark cell, as manifested by light microscopy (Setlow, 2003, 2013; Moir, 2006; Figure 1A). Germination is traditionally considered to occur without the need for any macromolecule synthesis (Steinberg et al., 1965; Vinter, 1970; Setlow, 2003, 2013; Moir, 2006); however, our recent results challenge this view, as we demonstrated that translation occurs during germination and is required for its execution (Sinai et al., 2015).

**Figure 1:**
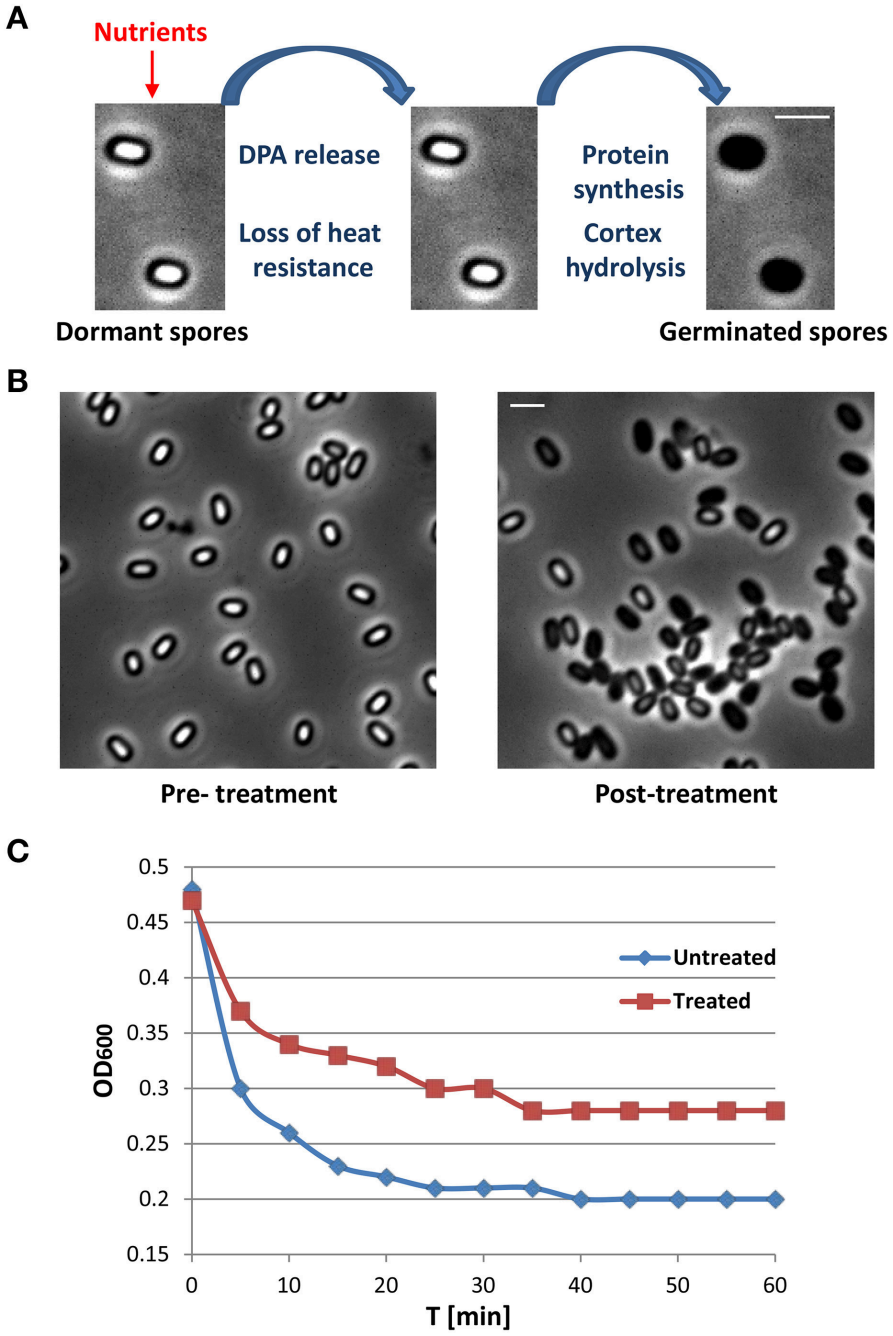
**(A)** Phase contrast images from time lapse microscopy of germinating *B. subtilis* spores. The different stages of germination are indicated. **(B)** Phase contrast images of a field of *B. subtilis* spores before (left: Pre-treatment) and after (right: Post-treatment) incubation for 18 h at 80°C. **(C)** Spores were incubated for 18 h at 80°C (treated), and then purified with Histodenz gradient as described by Korza et al. (2016). Untreated spores (untreated) were heat activated for 30 min at 75°C. Next, spores from both conditions were induced to germinate in the presence of L-alanine (*t* = 0), and germination was followed by decrease in OD_600_. Scale bars 1 μm.

Utilizing *Bacillus subtilis* (*B. subtilis*) as a model organism, we provided several lines of evidence supporting this view:

1. Protein synthesis was directly detected during germination by BONCAT (BioOrthogonal Non-Canonical Amino-acid Tagging) protein tagging technique. BONCAT allows the specific labeling and identification of newly translated proteins due to incorporation of a methionine analog (Dieterich, 2007). Protein synthesis was monitored when spores were induced to germinate with classical germinants such as L-alanine, or with the “non-nutrient” germinant Ca-DPA, in the absence of any other nutrients. Using this approach, we identified a core of 30 proteins, synthesized during this early stage, and thus defined the germination proteome (Sinai et al., 2015: Figure 4; Table S5).
2. Germination was halted when spores were pre-incubated with the translational inhibitors lincomycin and tetracycline, as indicated by the stalling of spores in their phase bright stage. Furthermore, BONCAT analysis of antibiotic-treated spores revealed a block in protein synthesis during germination, substantiating a direct effect of the antibiotics on protein synthesis (Sinai et al., 2015: Figure 4; Table S3).
3. Protein synthesis during germination was observed in real time. We investigated the production of MalS-GFP fusion by time lapse microscopy, as MalS is one of the earliest proteins produced in germinating spores. We showed that MalS-GFP fusion is translated in phase bright spores, indicating that germination was still in progress. These results were further corroborated by western blot analysis (Sinai et al., 2015: Figure 6; Figure S3).
4. Knockout of the genes encoding the translational factors RpmE and Tig, which are part of the germination proteome, largely delayed germination, without affecting vegetative growth, sporulation or the levels of key germination proteins. Moreover, we have shown by BONCAT, time lapse microscopy with MalS-GFP, and western blot analysis that knockout of *rpmE* and *tig* directly attenuates protein synthesis during germination (Sinai et al., 2015: Figures 5–6; Figure S2).

Importantly, we specifically delineated the sub-stage of germination at which protein synthesis becomes essential. We conducted DPA release and heat sensitivity assays on germinating antibiotic-treated spores as well as on Δ*tig* and Δ*rpmE* mutant spores. We found that these spores become sensitive to heat and release DPA with similar kinetics to that of untreated/wild type spores (Sinai et al., 2015: Figures 4B, 5C; Table S4), demonstrating that they initiated germination normally and were subsequently stalled.

A recent paper published by the Setlow laboratory (Korza et al., 2016) presumably challenges our findings, claiming that protein synthesis is not required for germination. This discrepancy was highlighted by a commentary written by Boone and Driks (2016), thoroughly comparing the two papers. Korza et al. based their observations on the exposure of dormant *Bacilli* spores to sub-lethal temperatures (75–80°C) for extended period of times (~20 h). Under such conditions, dormant spores lose much of their detectable rRNA, and therefore the authors argue that they are incapable of synthesizing proteins during germination. Furthermore, the authors present experiments claiming that these rRNA depleted spores are viable, and “germinate as well or better than spores with normal rRNA levels”. We claim here that the data presented in this manuscript do not support this statement for the following reasons:

1. The authors assume that there is a direct correlation between the rRNA levels within dormant spores and protein synthesis during germination. However, nowhere in this manuscript do the authors present an assay that directly measures ribosomal activity or protein synthesis. Further, the authors did not use any of our already established assays for monitoring protein synthesis during germination (antibiotics, BONCAT, MalS-GFP reporter, etc.).
2. The authors discard the possibility that rRNAs, similarly to proteins, can be rapidly synthesized during germination. Furthermore, we have previously clearly shown that incubation of spores at 50°C for 6 days resulted in the production of spores with reduced rRNA levels. However, upon exposure to germinants, these spores re-synthesized rRNA within minutes, at a time corresponding to germination, as indicated by sensitive and quantified microfluidic gel analysis (Segev et al., 2013: Figure 2C). These results were not referred to by the authors.

Moreover, the authors used ethidium bromide gels for rRNA detection. These less sensitive gels were used to argue for the absence of rRNA (without any quantification). However, even with these methodological weaknesses, when we closely examined and quantified their data, we could clearly see an increase in rRNA levels at time points corresponding to germination [Figure 8B (5 min) for *B. megaterium*; Figure 9B (30 min) for *B. subtilis*. The latter is apparent upon proper scaling and quantification of the gel image, data not shown] (Korza et al., 2016).

(3) One of the strongest conclusions of this manuscript is that heat-treated spores “can germinate as well as, or even better, than spores with normal rRNA levels”. This claim is highlighted by the authors to indicate that protein synthesis is not required for germination (again an indirect conclusion). In our opinion, this conclusion is not supported by the data presented for the following reasons:
  - The authors used DPA release as a measure of germination (Korza et al., 2016: Figures 5–6). However, although not indicated by the authors, we have shown that DPA release precedes and is independent of protein synthesis (Sinai et al., 2015). Thus, the use of this assay for probing germination, and specifically for demonstrating involvement of protein synthesis in germination, is misleading.
  - The authors used optical density to measure germination and outgrowth kinetics. Unfortunately, they did not use this assay to measure germination *per se* (by using medium that is supplemented with germinates but lacking nutrients). Nevertheless, even under these conditions their data show an obvious delay in both germination and outgrowth kinetics for *B. subtilis* (Korza et al., 2016: Figure 7B), and less obvious but still detectable for *B. megaterium* (Korza et al., 2016: Figure 7A; approximately 40% of the spores did not germinate before the initiation of outgrowth). Disappointingly, this crucial data has not been thoroughly discussed, and the authors even claim that “germination of *B. subtilis* spores was also faster than untreated spores”, in contrast to their own results.

We have repeated the heat treatment of *B. subtilis* spores under the same conditions as the authors (18 h at 80°C). We found that approximately 70% of the spores did not survive this treatment, as indicated by the loss of their phase bright appearance (Figure 1B). This signifies that the experiments performed by the authors referred to a small sub-population of spores that survived the harsh heat-treatment, while our previous data are based on assessment of the entire spore population without any bias.

Nevertheless, we have followed the purification step conducted by the authors to isolate viable spores and assayed their ability to germinate using optical density (and not DPA release). We found that 60 min post-induction of germination, only 65% of the purified spores could germinate, while 99% of the untreated spores completed germination after 25 min (Figure 1C).

In summary, the study conducted by Korza et al. (2016) does not support the claims and the conclusions made by the authors, and we would like to share our view with the readers.

## Author contributions

All authors listed, have made substantial, direct and intellectual contribution to the work, and approved it for publication.

## Funding

This work was supported by the European Research Council Advance Grant (339984) awarded to SBY.

### Conflict of interest statement

The authors declare that the research was conducted in the absence of any commercial or financial relationships that could be construed as a potential conflict of interest.

## References

[B1] Boone T. Driks A. (2016). Protein synthesis during germination: shedding new light on a classical question. J. Bacteriol. 198, 3251–3253. 10.1128/JB.00721-16 27736794 PMC5116928

[B2] Dieterich D. C. Lee J. J. Link A. J. Graumann J. Tirrell D. A. Schuman E. M. (2007). Labeling, detection and identification of newly synthesized proteomes with bioorthogonal non-canonical amino-acid tagging. Nat. Protoc. 2, 532–540. 10.1038/nprot.2007.52 17406607

[B3] Korza G. Setlow B. Rao L. Li Q. Setlow P. (2016). Changes in *Bacillus* spore small molecules, rRNA, germination and outgrowth after extended sub-lethal exposure to various temperatures: evidence that protein synthesis is not essential for spore germination. J. Bacteriol. 198, 3254–3264. 10.1128/JB.00583-16 27645383 PMC5116929

[B4] Moir A. (2006). How do spores germinate? J. Appl. Microbiol. 101, 526–530. 10.1111/j.1365-2672.2006.02885.x 16907803

[B5] Segev E. Rosenberg A. Mamou G. Sinai L. Ben-Yehuda S. (2013). Molecular kinetics of reviving bacterial spores. J. Bacteriol. 195, 1875–1882. 10.1128/JB.00093-13 23417486 PMC3624579

[B6] Setlow P. (2003). Spore germination. Curr. Opin. Microbiol. 6, 550–556. 10.1016/j.mib.2003.10.001 14662349

[B7] Setlow P. (2013). Summer meeting 2013-when the sleepers wake: the germination of spores of *Bacillus* species. J. Appl. Microbiol. 115, 1251–1268. 10.1111/jam.12343 24102780

[B8] Sinai L. Rosenberg A. Smith Y. Segev E. Ben-Yehuda S. (2015). The molecular timeline of a reviving bacterial spore. Mol. Cell 57, 695–707. 10.1016/j.molcel.2014.12.019 25661487 PMC4339302

[B9] Steinberg W. Halvorso H. Keynan A. Weinberg E. (1965). Timing of protein synthesis during germination and outgrowth of spores of *Bacillus cereus* strain T. Nature 208, 710–712. 10.1038/208710a0

[B10] Stragier P. Losick R. (1996). Molecular genetics of sporulation in *Bacillus subtilis*. Annu. Rev. Genet. 30, 297–241. 10.1146/annurev.genet.30.1.297 8982457

[B11] Vinter V. (1970). Symposium on bacterial spores: V. germination and outgrowth: effect of inhibitors. J. Appl. Bacteriol. 33, 50–59. 10.1111/j.1365-2672.1970.tb05233.x 5447474

